# Imidacloprid Alters Foraging and Decreases Bee Avoidance of Predators

**DOI:** 10.1371/journal.pone.0102725

**Published:** 2014-07-15

**Authors:** Ken Tan, Weiwen Chen, Shihao Dong, Xiwen Liu, Yuchong Wang, James C. Nieh

**Affiliations:** 1 Key Laboratory of Tropical Forest Ecology, Xishuangbanna Tropical Botanical Garden, Chinese Academy of Science, Kunming, Yunnan Province, China; 2 Eastern Bee Research Institute, Yunnan Agricultural University, Heilongtan, Kunming, Yunnan Province, China; 3 Division of Biological Sciences, Section of Ecology, Behavior, and Evolution, University of California San Diego, La Jolla, California, United States of America; French National Institute for Agricultural Research (INRA), France

## Abstract

Concern is growing over the effects of neonicotinoid pesticides, which can impair honey bee cognition. We provide the first demonstration that sublethal concentrations of imidacloprid can harm honey bee decision-making about danger by significantly increasing the probability of a bee visiting a dangerous food source. *Apis cerana* is a native bee that is an important pollinator of agricultural crops and native plants in Asia. When foraging on nectar containing 40 µg/L (34 ppb) imidacloprid, honey bees (*Apis cerana*) showed no aversion to a feeder with a hornet predator, and 1.8 fold more bees chose the dangerous feeder as compared to control bees. Control bees exhibited significant predator avoidance. We also give the first evidence that foraging by *A. cerana* workers can be inhibited by sublethal concentrations of the pesticide, imidacloprid, which is widely used in Asia. Compared to bees collecting uncontaminated nectar, 23% fewer foragers returned to collect the nectar with 40 µg/L imidacloprid. Bees that did return respectively collected 46% and 63% less nectar containing 20 µg/L and 40 µg/L imidacloprid. These results suggest that the effects of neonicotinoids on honey bee decision-making and other advanced cognitive functions should be explored. Moreover, research should extend beyond the classic model, the European honey bee (*A. mellifera*), to other important bee species.

## Introduction

Bees play an important global role as pollinators of native plants and crops [1], [2]. However, pollinator foraging behavior, and thus the ability of pollinators to pollinate, can be negatively influenced by pesticides at sublethal doses [3]. Impaired foraging may reduce colony fitness [4], contributing to bee population declines [5]. Research has focused on neonicotinoid pesticides, a class of compounds that became commercially available in the early 1990’s [6] and which bind to insect cholinergic receptors, causing death at sufficient concentrations [7]. In fact, the European Union recently restricted the use of neonicotinoids because of concerns about their effects on bees [8]. However, neonicotinoids remain widely used around the world [9]. Continued research on the sublethal effects of these compounds is therefore important because assays that only test for lethality, the standard approach for determining safe doses, do not reveal how these compounds impair bee behaviors that are intimately involved in pollination and colony fitness [3], [10], [11].

Imidacloprid is a neonicotinoid pesticide: its metabolites are nicotinic acetylcholine receptor (nAChR) agonists in honey bee neurons [12] and therefore have widespread behavioral consequences. Field-realistic levels of imidacloprid in nectar reduce honey bee performance by 6–20%: decreasing hive entrance activity [13], learning ability [14], food uptake [15], and locomotion [16]. Imidacloprid also decreases foraging activity [17] and the ability of bees to successfully return to the nest [18], perhaps because of navigational deficits [3].

Such cognitive impairments are particularly intriguing because honey bees have sophisticated decision-making skills [19], and the deficits elicited by neonicotinoids may therefore be complex and, perhaps, unexpected. For example, sublethal doses of imidacloprid reduce olfactory [13], [20], [21] and visual [22] learning. Imidacloprid also evidently alters bee internal thresholds, elevating the sucrose response threshold and reducing waggle dancing even for very rich nectar sources [16], [23]. However, the role of imidacloprid in altering other types of decision-making remains poorly understood.

In addition to deciding whether a nectar source is sweet enough to collect or recruit for, bees also evaluate and respond to the risk of predation during foraging [24]. Bees face a wide variety of predator threats and can normally avoid dangerous food sources. For example, honey bees avoid flowers with living crab spiders [25] and live mantises [26]. In Asia, hornets within the genus *Vespa* are major honey bee predators [27], [28], and the hornet *V. velutina* attacks *A. cerana* colonies [29]–[31]. Recently, we showed that this hornet species will capture *A. cerana* foraging on flowers [32]. *Apis cerana* foragers consequently show a strong aversion to a feeder with a *V. velutina* hornet and will reduce visitation to such a dangerous feeder by 78% but will not reduce visitation to a feeder with a harmless butterfly [32]. Given that neonicotinoids can alter *A. mellifera* foraging behavior [15], we hypothesized that neonicotinoids can also impair a bee’s judgment about danger, altering its ability or its willingness to avoid predators.

The sublethal effects of neonicotinoids have been studied in relatively few bee species, and comparatively little is known about neonicotinoid effects on native bees in large areas of the world such as Asia. Studies have demonstrated detrimental sublethal effects of neonicotinoids in European honey bees (*A. mellifera*), European bumble bees (*Bombus terrestris*), and the South American stingless bee (*Melipona quadrifasciata anthidioides*) [3], [4], [33], [34]. Recently, Arena et al. [35] conducted a meta-analysis and suggested that native Asian honey bees species (*A. cerana* and *A. florea*) may have a higher sensitivity to pesticides than *A. mellifera*. However, to the best of our knowledge, no published studies have systematically compared the effects of neonicotinoids on the foraging behaviors of multiple honey bee species. Such knowledge would be valuable. For example, the native Asian honey bee, *A. cerana,* plays a large role in agriculture [36], [37], and is an important native pollinator of native Asian plants [37], [38]. *Apis cerana* is widespread and is found throughout southern and eastern Asian, extending from India to China [39]. In China alone, more than two million managed colonies of *A. cerana* are used for honey production and crop pollination [37]. Previous studies have shown that pesticides such as carbamates [40], [41], synthetic pyrethroids [42], and imidacloprid [43], [44] are toxic to *Apis cerana.* Imidacloprid can bind to two different receptor sites within *A. cerana* nAChRs [45]. However, studies have not explored the sublethal effects of neonicotinoids on *A. cerana* even though imidacloprid is widely used in China [46]. We therefore tested the effects of field-realistic doses of imidacloprid on *A. cerana* foraging behavior and decision-making with respect to food source danger, the hornet *V. velutina,* a native Asian predator and an emerging threat to *A. mellifera* in Europe [47].

## Materials and Methods

We conducted experiments from October to November 2013. This field season corresponds to the time of peak hornet activity, when *V. velutina* actively hunts honey bees at our field site Yunnan Agricultural University, Kunming, China (22°42′30 N, 100°56′01 E, 1890 m altitude). Our field season also corresponded to a period of floral dearth, which facilitated feeder training of bees. We used three colonies of *A. cerana*, each with four frames of bees and brood.

### Imidacloprid concentrations

The scientific literature commonly uses four different units (µg/L, nmol/L, µg/Kg, and ppb) to express neonicotinoid concentrations. To facilitate comparisons, we provide conversions for all of these commonly used values in our figures and in our discussion, as appropriate. For conversions involving sucrose concentrations and density (at 20°C), we use tables published in Bubnik et al. [48]. In our Discussion, we convert concentration values reported by the original study to a common unit (µg/L) based upon the sucrose concentrations used in the original study) and give the value reported by the original study in parentheses.

**Figure 1:**
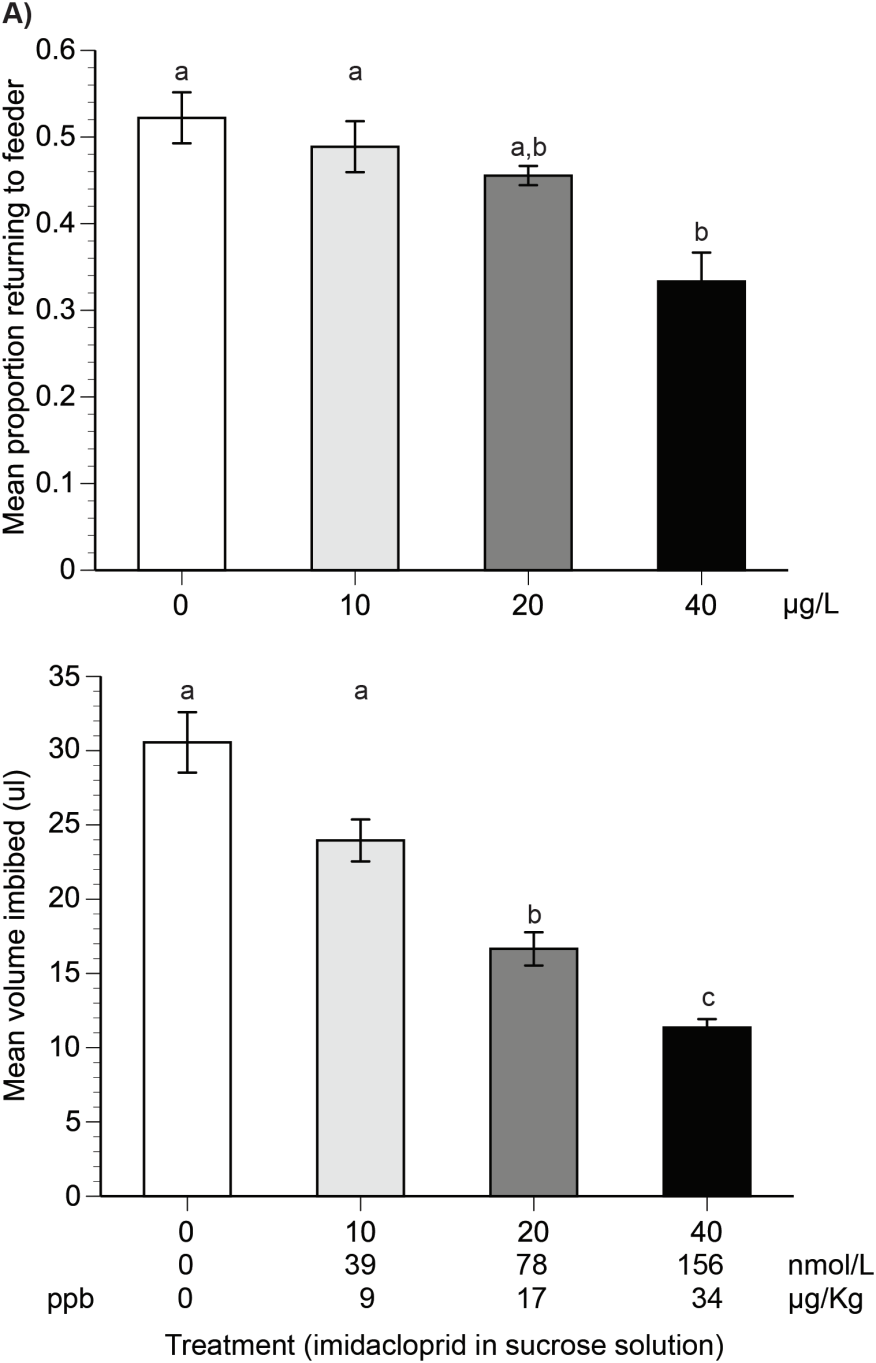
Effect of imidacloprid treatments on bee return rates and nectar collection (imbibing) volume. a) The mean proportion of trained bees that returned to the test feeders with different imidacloprid treatments. b) The mean volume of 1.25 M sucrose solution collected by bees trained to a safe feeder with no hornet predators. Standard error bars are shown. Different letters indicate treatments that are significantly different from each other (Tukey HSD tests, *P*<0.05). The imidacloprid concentrations are given in commonly used units, with ppb and µg/Kg shown in the same row because the numeric values are identical. Different shades of gray correspond to different imidacloprid concentrations. Sample sizes are given in the Methods.

We used 1.25 M sucrose solutions (37% sucrose w/w) with field-realistic doses of imidacloprid [6]: 10 µg/L (8.6 parts per billion), 20 µg/L (17.2 ppb), and 40 µg/L (34.0 ppb) of imidacloprid, corresponding to 39.1 nmol/L, 78.2 nmol/L, and 156.5 nmol/L (Fig. 1). We chose these concentrations to cover a range of field-realistic, sub-lethal doses. Imidacloprid residues have been found to occur at 1–50 ppb in the nectar and pollen of a variety of crop species [6]. In citrus trees treated with imidacloprid and grown within an enclosure, residues of 3–39 µg/L were detected in nectar, with an average of 16.4 µg/L from floral nectar and 15.3 µg/L from bee crops (caged tunnel studies) [49]. In open field studies, total residues of imidacloprid were 5.0 µg/L in floral nectar and 3.5 µg/L in bee crops [49]. Field realistic doses of imidacloprid from a variety of crops and studies are 0.7–10 µg/L, corresponding to a 0.024–0.3 ng dose per nectar load [50].

The imidacloprid concentrations that we used (39.1–156.5 nmol/L) were sublethal. In *A. mellifera*, only imidacloprid concentrations ≥1000 nmol/L increased mortality: 10 and 100 nmol/L did not alter mortality [51]. The acute 48-hr oral LD_50_ for imidacloprid ranges widely and depends upon a variety of factors that include bee physiological condition and season [14], [24], [52]. Values range between 3.7 ng/bee [53] and >81 ng/bee [54]. The average nectar load at our highest treatment concentration (40 µg/L) contained a sublethal dose (0.52 ng of imidacloprid/bee). None of the foragers died from exposure to our imidacloprid treatments during the experiments.

### Training bees

We used three colonies in all experiments. We trained bees by capturing departing foragers at the hive entrance in a vial and releasing them slowly at the training feeder placed 130 m from the focal colony. The feeder consisted of a 70 mL vial (8 cm high) with 18 holes (each 3 mm diameter) drilled around its lid. Once it was filled with sucrose solution, the vial was inverted on a blue plastic square and bees could slowly collect sugar solution through the drilled holes [32]. We filled this feeder with unscented 1.25 M sucrose solution (37% sucrose w/w). We marked all trained foragers with individually numbered honey bee queen tags attached to their thoraces with resin. We used a different set of bees for each of the following three experiments.

### Foraging experiment

We trained bees to the unscented 1.25 M sucrose feeder (see above). After bees made 10 trips to the training feeder, it was replaced with an identical feeder containing one of four different treatment concentrations of imidacloprid (0, 10, 20, and 40 µg/L) in 1.25 M sucrose. We allowed the bees to sample the treatment feeder and then recorded which bees subsequently returned to this feeder. A bee that made a single return within 1 hr was counted as a returning bee. The proportion of bees that continued to forage at the different imidacloprid concentrations was then calculated. In total, we trained 360 bees, 120 from each colony, and 90 bees per treatment.

### Nectar collection experiment

To determine the amount of the treatment solutions that bees collected, we trained bees as before. After 10 training trips, we changed the feeder to one containing one of the four treatment concentrations of imidacloprid. Each bee was allowed to visit the treatment feeder 10 times so that its collection volume would reach equilibrium. After the 10^th^ visit, the bee was captured at the nest upon its return. We used CO_2_ to anesthetize each bee, weighed it, gently squeezed its abdomen and absorbed the contents of its collected nectar onto tissue paper, and then immediately weighed it again to determine the mass of nectar it had collected. The volume collected per bee was then calculated. In total, we used 84 bees, 28 from each colony, and 21 bees per treatment.

### Danger experiment

To test the effect of imidacloprid on bee decision making about danger, we trained bees as previously described. Once the marked bees made 10 consecutive visits to the training feeder, we surrounded this feeder with a cage (70×66×52 cm) with a single exit and entrance. The cage allowed us to restrict bee access and to ensure independent choices by testing individual choices in the absence of other bees. Bees were exposed to the imidacloprid once the training feeder was placed inside the foraging cage. Each forager was randomly assigned to one imidacloprid treatment concentration and was exposed to this concentration for 10 collecting visits before we tested its choice of safe vs. dangerous feeders.

After the bees had learned to forage inside the cage for these 10 successive visits, we randomly selected seven bees from each colony for the experiment. We replaced the single feeder with an array of two identical unscented 1.25 M sucrose feeders spaced 40 cm apart inside the cage. These feeders both contained the same treatment solution. For example, if a bee was trained to a 40 µg/L imidacloprid solution, the safe and dangerous feeders also contained this solution. To create a “dangerous” feeder, we captured *V. velutina* hornets with insect nets and tethered a single hornet 10 cm above the feeder with a stiff wire wrapped between the thorax and abdomen. The “safe” feeder had a similar wire, but with no hornet. Tan et al [32] demonstrated that a significant majority of bees exposed to such a feeder array consistently chose the safe feeder.

We monitored five successive choices of our trained bees. Thus, each bee was exposed over approximately 1.5 hrs for 15 total visits to a given treatment. After each visit, we replaced the feeders to eliminate potential odor marks and randomly swapped dangerous and safe feeder positions to avoid potential site bias. In total, we used 80 bees (28 bees from colony 1, 28 bees from colony 2, and 24 bees from colony 3), 20 bees per treatment.

### Statistics

We arcsin-square-root transformed the proportion of visiting bees, log-transformed the volume of sucrose collected, and used Analysis of Variance (ANOVA) to test for a significant effect of treatment, with Tukey Honestly Significant Difference (HSD) post-hoc tests to compare between treatments [55]. These data met parametric assumptions as determined by residual analyses. We used Chi-square tests to compare observed and expected bee choices. For each bee, we calculated the proportion of visits to the safe feeder and classified the bee as preferring the safe feeder if it chose the safe feeder ≥60% out of its five trips. To compare overall choices, we used a null hypothesis expectation of 50% of bees visiting each feeder. Finally, we used Chi-square tests to compare the choices of imidacloprid-treated bees against the choices of control bees. We used a Repeated-Measures Analysis of Variance (ANOVA) model with individual bee (random effect) nested within treatment to test the effect of two fixed effects, treatment and trial number (the visit number of each bee for the five consecutive visits tested), on bee choices (0 = dangerous feeder, 1 = safe feeder). We included colony identity as a random variable in the ANOVA models.

### Ethics statement

This research was conducted in full compliance with the laws of the People’s Republic of China. No specific permits were required for our field studies, which were conducted at Yunnan Agricultural University. Our studied involved colonies of *A. cerana* and hornets, *V. velutina*. Neither species is endangered or protected.

## Results

### Foraging experiment

There is a significant effect of pesticide concentration on the proportion of trained bees that returned to the feeder (*F*_3,6_ = 7.67, *P* = 0.018), with colony accounting for <1% of model variance. As compared to the control and the 10 µg/L feeders, significantly fewer bees (23% fewer) returned to the 40 µg/L feeder. There is no significant difference between control and 10 µg/L bees. There is no significant difference between 20 µg/L and 40 µg/L bees (Tukey HSD test, Fig. 1A).

### Nectar collection experiment

Increasing pesticide concentration significantly decreases the amount of sucrose solution that bees collect (*F*_3,78_ = 45.4, *P*<0.0001, Fig. 1B). Colony accounts for 2% of model variance. A Tukey HSD test shows that bees significantly decreased the average volume collected by 46% and 63% for 20 µg/L and 40 µg/L imidacloprid solutions, respectively, as compared to the control solution. There is no significant difference between the volume collected from the control and 10 µg/L solutions (Tukey HSD test, Fig. 1B). Based upon the average nectar volume collected per trip, each bee collected (but did not necessarily absorb into its hemolymph) 0.27, 0.39, and 0.52 ng of imidacloprid per trip from solutions with imidacloprid concentrations of 10, 20, and 40 µg/L, respectively. However, these values likely do not represent the amount of pesticide that bees individually absorbed into their bodies per trip since the majority of this sugar solution was regurgitated to other bees and stored inside the nest.

### Danger experiment

**Figure 2:**
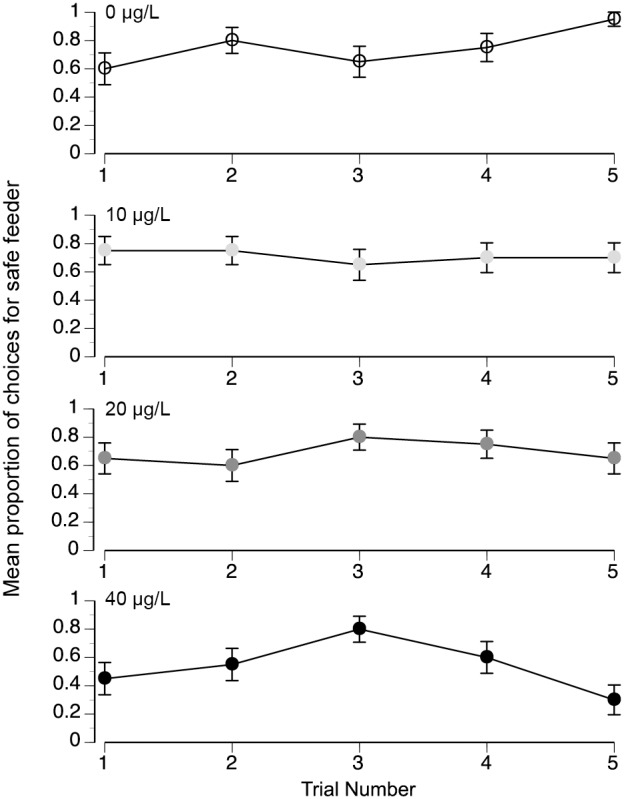
Mean proportion of choices for the safe feeder over five trials. The different treatments are identified above each plot (1 = all choices for safe feeder). Different shades of gray correspond to different imidacloprid concentrations. Standard error bars are shown.

In the danger experiment, there is a significant effect of treatment (*F*_3,74_ = 3.57, *P* = 0.03) but no significant effect of trial (*F*_1,319_ = 0.40, *P* = 0.53) and no significant interaction between treatment and trial (*F*_3,316_ = 1.63, *P* = 0.18). Colony and individual bee account for respectively 0.5% and 7% of model variance. Individual foragers therefore exhibited preferences that did not significantly alter over multiple trials (Fig. 2).

**Figure 3:**
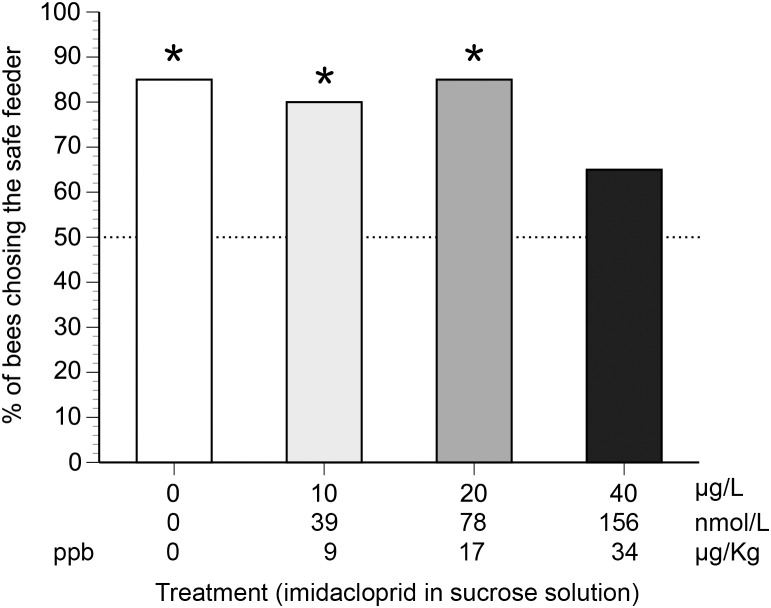
The effect of imidacloprid on the percentage of bees choosing a safe over a dangerous feeder. Stars above bars indicate treatments in which bees significantly avoided the dangerous feeder (*P*<0.05). Different shades of gray correspond to different imidacloprid concentrations. A dashed line shows the null hypothesis expectation: 50% of bees choose the safe feeder.

Control bees that received no imidacloprid avoided the hornet: overall, 85% of control bees chose the feeder without the hornet (χ^2^_1_ = 9.8, *P* = 0.002). Similarly, bees that received 10 µg/L (χ^2^_1_ = 7.2, *P* = 0.007) or 20 µg/L (χ^2^_1_ = 9.8, *P* = 0.002) also avoided the hornet: overall, 80% and 85% respectively chose the feeder without the hornet. However, bees that received the highest dose (40 µg/L) did not avoid the hornet: only 65% chose the feeder without the hornet, not significantly different from a random choice (χ^2^_1_ = 1.8, *P* = 0.18, Fig. 3).

We then compared the distribution of bee choices to the observed distribution of control bee choices. Bees treated with the highest level of imidacloprid (40 µg/L) exhibited significantly different choices than control bees (χ^2^_1_ = 6.3, *P* = 0.012). Bees treated with the lower imidacloprid levels of 10 µg/L (χ^2^_1_ = 0.4, *P* = 0.53) and 20 µg/L (χ^2^_1_ = 0, *P* = 1.0, observed and expected distributions were identical) did not make choices that were significantly different from control bees (Fig. 3).

## Discussion

Neonicotinoid pesticides can impair honey bee cognitive abilities [52]. We provide the first demonstration that sublethal concentrations of a neonicotinoid, imidacloprid, can also impair honey bee decision-making about danger, significantly increasing the likelihood that a bee will visit a dangerous feeder with a hornet predator. At an imidacloprid nectar concentration of 40 µg/L (34 ppb), 1.8 fold more bees chose the dangerous feeder as compared to bees fed with no imidacloprid (Fig. 3). These preferences were consistent and did not significantly vary over multiple choices by the same bees (Fig. 2). In addition, we provide the first data demonstrating that foraging by *A. cerana*, a native bee and important pollinator of agricultural crops and native plants in Asia, can be influenced by sublethal doses of imidacloprid, which is also used widely in Asia. Imidacloprid reduced food collection: 23% fewer foragers returning to collect the 40 µg/L imidacloprid-laced nectar as compared with the control nectar. Compared to controls, bees that returned to the feeder respectively collected 46% and 63% less nectar containing 20 µg/L and 40 µg/L imidacloprid. These effects could arise if imidacloprid reduces bee motivation to forage, reduces their physical ability to imbibe (drink) nectar, or both.

### Effects on visitation and nectar collection

The reduction in feeder visitation and collecting that we observed (Fig. 1) matches what other studies have found for the closely related European honey bee, *A. mellifera*. Imidacloprid at 7 µg/L (6 µg/kg) reduced *A. mellifera* foraging at a 1.38 M sucrose solution (40% w/w) [56]. In our experiment, *A. cerana* foragers were somewhat more resistant, and visitation for our 37% w/w sucrose solution did not significantly decline until we provided a concentration of 40 µg/L (Fig. 1A). Our result is a better match for the data of Yang et al [57], who showed that *A. mellifera* foragers significantly delayed their willingness to return to a 50% sucrose solution w/w containing imidacloprid at 50 µg/L (close to 40 µg/L). Similarly, a higher concentration of imidacloprid, 145 µg/L (115 ppb), reduced *A. mellifera* visits to a 2 M (55% w/w) sucrose feeder by 47% as compared to controls [17]. In our foraging experiment, we tested bees immediately after exposing them to imidacloprid. Thus, it is possible that their aversion was based upon their gustatory detection of the contaminant, not upon its neurological effects. However, our *A. cerana* foragers nonetheless exhibited the reduced visitation shown in *A. mellifera*.

In the collection experiment, we allowed each *A. cerana* forager to visit the treatment feeder 10 times in order to equilibrate its response to the treatment solution. These *A. cerana* foragers significantly decreased by 43% the volume of 20 µg/L imidacloprid solution (37% sucrose w/w) that they collected as compared to controls (Fig. 1B). This is similar to what is reported for *A. mellifera* foragers, which decreased the volume of 50% sucrose solution that they collected by 69% when it contained 29 µg/L (24 µg/Kg) of imidacloprid [13]. We note that the behavioral differences observed between these studies may arise from differences in bee species, sugar concentrations, and pesticides doses. For example, bees may tolerate a higher concentration of contaminants in sweeter nectar. Different environmental conditions may also play a role [3]. However, imidacloprid generally reduces the volume of sucrose solution collected by both species.

The overall exposure of each colony to imidacloprid was minimal. Based upon the number of bees trained and their visits to each treatment concentration in all three experiments over two months, each colony received only 3.6 ng of imidacloprid per day, on average. This dose was potentially shared among thousands of bees per colony. Control bees were evidently not affected by this potential exposure (Figs. 1 & 3).

### Imidacloprid exposure in the danger experiment

By the time they were tested in the danger experiment, each bee had made 15 visits over approximately 1.5 hrs, sufficient time for imidacloprid absorption. For example, 20 min [17] or 60 min [23] is sufficient for orally administered imidacloprid to elicit strong effects: reduction in foraging activity and longer foraging flights [17] and reduced waggle dancing [23]. Because we allowed our bees to unload their collected sucrose solution to nestmates, it is unclear how much imidacloprid they absorbed before exchanging their collected nectar with nestmates. If bees absorbed a full nectar load over the 15 visits, each bee would have been exposed, on average, to 0.27, 0.39, and 0.52 ng of imidacloprid. These doses are based upon the average collection volume of the different imidacloprid concentrations (Fig. 1B) and therefore reflect decreased collection at higher imidacloprid concentrations. In addition, the tested bees completed all 15 visits to the feeder and were therefore exposed for the same number of trips. Thus, after compensating for reduced collection of higher imidacloprid concentrations, bees were exposed to 1.4 and 1.9 fold higher doses of imidacloprid at the 20 µg/L and 40 µg/L treatments as compared to the 10 µg/L treatment. We note that the effects of imidacloprid can be complex due to separate actions of imidacloprid and its toxic metabolites, 5-hydroxyimidacloprid and olefin [52]. In *A. mellifera*, imidacloprid has a metabolic half life of 4.5–5 hrs [58], and thus, over the average 1.5 hr exposure period of our experiment, imidacloprid was likely the main molecule responsible for altering bee behavior.

### Effects on decision-making about danger

*Apis cerana* foragers that were not exposed to imidacloprid stayed clear of the dangerous feeder with the *V. velutina* hornet, in agreement with previous research [32]. However, the foragers that continued to visit the 40 µg/L feeder showed no significant avoidance of the dangerous feeder (Fig. 3). Did the imidacloprid-treated bees (1) suffer from a decision-making deficit, (2) were they unable to sense the predator, or (3) were they unable to control their flight and therefore randomly landed on the dangerous feeder? The latter two explanations seem unlikely. Although imidacloprid can affect visual learning in honey bees [22], there is no evidence that it degrades vision at sublethal doses. Moreover, the hornets are quite large (averaging 2 cm in length) and have visually conspicuous aposematic coloration [29]. In addition, the foragers collecting 40 µg/L imidacloprid were still able to navigate between feeder and nest, capably orienting towards a feeder that was only 8 cm tall. This task requires good vision. The bees flew in a straight line and did not exhibit any signs of tremors as they collected the sucrose solution. At the nest, these foragers also found and unloaded their collected nectar without problems for five successive trips. Interestingly, the bee foragers that continued to visit even the highest concentrations of imidacloprid would, in other studies, have been considered bees that were relatively unaffected by the treatment because they continued to forage. However, in our experiment, we are able to demonstrate impairment: these bees did not distinguish the dangerous from the safe feeder. Whether this lack of discrimination arises from a decrease in “fear” of the hornet, an inability to make appropriate decisions, or from some other cognitive deficit remains unclear, but is a fascinating area for future research.

In general, we know relatively little about the effects of pesticides on bee information processing. Schricker et al. [59] hypothesized that bees sublethally poisoned with the organophosphate, parathion, had degraded integration of information in their central nervous system. Belzunces [52] suggested that pesticide exposure may lead to incorrect interpretation of external stimuli. Eiri and Nieh [23] proposed that imidacloprid can alter honey bee decision-making by reducing the number of recruitment dance circuits that foragers perform for a good food source. Bees increase the number of waggle dance circuits that they perform according to how valuable they consider a resource, and their perception of resource value can be altered by a neuromodulator, octopamine [60]. Neonicotinoids may also alter bee perceptions about food. Eiri and Nieh [23] found that a single imidacloprid dose of 0.21 ng/bee (24 ppb) resulted in bees that continued to visit a rich 2.0 M (55% w/w) sucrose solution 24 hours later. These bees did not exhibit any impairments in flight, walking, or waggle dancing, a task requiring complex coordination [61]. Treated bees simply performed fewer dance circuits than controls, suggesting that they perceived the food, which was pure and free of any imidacloprid, as being less valuable for the colony [23].

Future research on sublethal doses of neonicotinoids may therefore reveal unsuspected effects on the complex decision-making shown by honey bees and contribute towards developing more sophisticated assays for determining safe application levels for neonicotinoids and future pesticides. Studies that also examine long term exposure or exposure at a sensitive developmental stage (such as in larvae [21]) would be beneficial. Fitness consequences of these impaired decisions should also be measured. For example, it is unclear if bees exhibiting risky behavior induced by imidacloprid suffer from increased predation. However, this possibility adds a new peril that deserves further study.
